# The role of her2-targeted therapies in women with her2-overexpressing metastatic breast cancer

**DOI:** 10.3747/co.v16i4.469

**Published:** 2009-08

**Authors:** S. Dent, Sh. Verma, J. Latreille, D. Rayson, M. Clemons, J. Mackey, Su. Verma, J. Lemieux, L. Provencher, S. Chia, B. Wang, K. Pritchard

**Affiliations:** * The Ottawa Hospital Cancer Centre and University of Ottawa, Ottawa, ON; † Réseau Cancer Montérégie and Université de Sherbrooke, Longueuil, QC; ‡ Atlantic Clinical Cancer Research Unit and QEII Cancer Care Program, Halifax, NS; § Princess Margaret Hospital, Toronto, ON; || Cross Cancer Institute, University of Alberta, Edmonton, AB; # Sunnybrook Odette Cancer Centre and University of Toronto, Toronto, ON; ‡‡ British Columbia Cancer Agency–Vancouver Centre, Vancouver, BC; §§ Remedy Communications Limited, Toronto, ON; ** Centre de Recherche du Centre Hospitalier affilié universitaire de Québec, Quebec City, QC; †† Hôpital du Saint-Sacrement, Quebec City, QC

**Keywords:** Metastatic breast cancer, trastuzumab, treatment beyond progression, re-treatment, her2-targeted therapy

## Abstract

The role of targeted therapies in the treatment of women with breast cancer has been rapidly evolving. Trastuzumab, a monoclonal antibody against the human epidermal growth factor receptor 2 (her2), was the first her2-targeted therapy that clearly demonstrated a significant clinical benefit for women with her2-overexpressing metastatic breast cancer (mbc). However, in recent years it has become increasingly apparent that, when trastuzumab is used in the first-line setting in combination with chemotherapy, most women eventually develop progressive disease. Determining the treatment options available to women who have progressed while on trastuzumab therapy has been hampered by a paucity of high-quality published data. In addition, with the standard use of trastuzumab in the adjuvant setting (for eligible her2-positive patients), the role of anti-her2 agents for patients who experience a breast cancer relapse has become a clinically relevant question. This manuscript reviews current available data and outlines suggestions from a panel of Canadian oncologists about the use of trastuzumab and other her2-targeted agents in two key mbc indications:

- Treatment for women with her2-positive mbc progressing on trastuzumab (that is, treatment beyond progression)
- Treatment for women with her2-positive mbc recurring following adjuvant trastuzumab (that is, re-treatment)

The suggestions set out here will continue to evolve as data and future trials with trastuzumab and other her2-targeted agents emerge.

## 1. INTRODUCTION

The human epidermal growth factor receptor 2 (her2) is a transmembrane tyrosine kinase receptor protein that is part of the her family of growth factor receptors (her1 to her4). The her2 receptor is involved in cell–cell and cell–stroma communication, primarily through signal transduction involving the Ras/mitogen-activated protein kinase and phosphatidylinositol 3 kinase (pi3k)/Akt pathways 1. These signals ultimately promote cell proliferation, survival, and motility. Amplification of the her2/*neu* gene and resulting overexpression of the her2 protein occurs in approximately 20% of invasive primary breast cancers 2. The her2 alteration in early-stage breast cancer is associated with more aggressive disease and a higher risk of relapse than is seen with her2-negative cancers 3–7.

Trastuzumab is a monoclonal antibody that binds to the extracellular domain of her2. Several mechanisms of action underlie the antitumour effects of trastuzumab. Trastuzumab blocks her2-activated cell signalling, thereby reducing cell proliferation and restoring ability to undergo apoptosis by inhibiting the pi3k/Akt pathway 1,8,9,a. The result is increased cellular sensitivity to chemotherapy and radiotherapy 10.

Trastuzumab has been shown to inhibit her2- regulated angiogenesis 8,11–13 and, in preclinical models, to recruit the immune system through antibody-dependent cellular cytotoxicity, triggering activation of natural killer cell–mediated apoptosis 14–16. Trastuzumab has also been shown to prevent the formation of p95her2 (a truncated active form of her2), which may lead to inhibition of tumour development 8,17.

In pivotal first-line metastatic breast cancer (mbc) trials (Table I), the addition of trastuzumab to standard systemic chemotherapy treatment resulted in significantly improved time to disease progression, improved response rates, and an overall survival benefit (as compared with chemotherapy alone) 18–20. Consequently, single-agent trastuzumab following chemotherapy or trastuzumab in combination with chemotherapy is now considered standard treatment for mbc patients who overexpress her2. In clinical practice, trastuzumab is usually continued until disease progression.

There is considerable controversy regarding the potential clinical benefit of continuing trastuzumab after relapse—that is, therapy beyond disease progression —as a component of second and subsequent lines of systemic therapy. As well, with the approval and widespread incorporation of trastuzumab as a component of adjuvant systemic therapy for her2-positive early-stage breast cancers (stages i–iii), uncertainty remains regarding the potential role of trastuzumab-based therapy upon relapse.

Data regarding these two clinical questions remain limited and primarily consist of nonrandomized retrospective (Level 3) evidence. The goal of the present paper is to review the current evidence and to outline suggestions from a panel of Canadian oncologists regarding the use of trastuzumab and other her2-targeted therapies in two key mbc patient indications:

- Treatment for women with her2-positive mbc progressing on trastuzumab (that is, treatment beyond progression)
- Treatment for women with her2-positive mbc recurring following adjuvant trastuzumab (that is, re-treatment)

## 2. DEVELOPMENT OF PANEL SUGGESTIONS

The authors of this paper met in Toronto for a one-day conference in June 2008. The panel reviewed results of the latest trials evaluating trastuzumab and other her2-targeted agents in the neoadjuvant, adjuvant, and metastatic settings. Based on trial information, suggestions were formulated for each setting. For mbc, a draft manuscript reviewing the current available data and outlining the suggestions from the panel was initially written by a medical writer (BW) and was reviewed and revised by four main panel members (SD, KP, ShV, and JL). The final manuscript was approved by the remaining seven panel members (JM, SuV, DR, SC, MC, JL, and LP). Published and presented clinical trials results (available as of March 2009) were incorporated into the present document. Support for the initial meeting of the Canadian advisory panel and the development of the present manuscript was provided by an unrestricted educational grant from Hoffmann–La Roche Canada. The authors received an honorarium for attending the meeting, but not for writing the manuscript. The authors are solely responsible for the content of the manuscript, with no restrictions set by the sponsor.

## 3. TRIALS IN MBC

Two major pivotal trials involving more than 650 women with her2-positive mbc have examined the role of trastuzumab in combination with systemic chemotherapy as compared with chemotherapy alone in the first-line setting 18–20. In both studies, although different chemotherapeutic regimens were used, the addition of trastuzumab, as compared with chemotherapy alone, significantly prolonged time to disease progression, increased response rate, prolonged duration of response, and increased overall survival benefit (Table I).

Several small, retrospective case–cohort studies have examined the role of continuing trastuzumab beyond progression in second- and subsequent-line therapy with various chemotherapy partners. Overall response rates ranged from 18% to 50% in the second line, 14.3% to 38.1% in the third line, 19.2% to 20% in the fourth line, and less than 10% in fifth-line trastuzumab-based regimens. Time to progression was reported as 4.0–9.0 months in the second line, 3.5–9 months in the third line, 4–4.9 months in the fourth line, and approximately 4 months in fifth-line trastuzumab-based regimens (Table II) 21–29.

Similar results of continuing trastuzumab with various chemotherapy partners beyond progression in women with mbc have been observed in prospective phase ii trials, although data remain limited. Across studies, overall response rates of 11%–50% were observed after a second-line trastuzumab-based regimen, with a greater than 60% response rate observed with one third-line trastuzumab-based regimen (Table III). Time to progression ranged from 6 months to 8 months in second-line and 9 months in third-line trastuzumab-based regimens (Table III) 30–36.

The French Hermine trial, a prospective observational study in women who had her2-positive mbc, contributed intriguing survival data in the setting of progression 37,38. A total of 623 patients were enrolled, of whom 221 (cohort A) received first-line trastuzumab-based therapy (that is, with paclitaxel, docetaxel, vinorelbine, or capecitabine) and 117 (cohort B) received trastuzumab-based therapy as part of second-line treatment 38. After 2 years of follow-up, significantly longer overall survival was observed in patients treated with first-line trastuzumab (cohort A) as compared with those who discontinued trastuzumab after initial progression (Table IV). For patients who continued trastuzumab as a component of second-line therapy (cohort B), overall survival from the first trastuzumab treatment was again longer than it was in patients who discontinued trastuzumab (Table IV). Given the nonrandomized nature of the study and the potential biases involved in the selection of patients who received second-line therapy, the foregoing results must be interpreted with caution. It is feasible that underlying patient characteristics could have contributed to the observed improvement in overall survival in patients who were able to continue to receive trastuzumab therapy at the time of disease progression.

### 3.1 Randomized Clinical Trials

A recently published study addressed the question of continuing trastuzumab beyond progression after first-line treatment^39^ (Table V). This prospective phase iii trial compared capecitabine plus trastuzumab with capecitabine alone in patients with locally advanced or metastatic breast cancer with disease progression during trastuzumab treatment. Patients could have received up to one prior chemotherapy regimen for mbc. Patients were required to be trastuzumab-free for fewer than 6 weeks, to have been taking trastuzumab for at least 12 weeks, and to have a baseline left ventricular ejection fraction of 50% or better. A total of 156 (of a planned 482) patients were accrued. During the recruitment phase of the study, lapatinib was registered by the U.S. Food and Drug Administration for the indications being tested, and on the advice of the Independent Data Monitoring Committee, this study was terminated prematurely. The primary objective was time to progression; secondary objectives were safety, objective response rates, clinical benefit rates, and overall survival 39. Patients received capecitabine (2500 mg/m^2^) for 14 days of a 21-day cycle, and trastuzumab (6 mg/kg) was administered in 3-week cycles. Compared with capecitabine alone, the addition of trastuzumab to capecitabine resulted in significant improvements in time to progression (8.2 months vs. 5.6 months; hazard ratio: 0.69; 95% confidence interval: 0.48 to 0.9; *p* = 0.0338), with significant improvements in overall response rates (48.1% vs. 27.0%, *p* = 0.0115) and clinical benefit rate (75.3% vs. 54.0%, *p* = 0.0068).

Median overall survival was not statistically different (25.5 months vs. 20.4 months, *p* = 0.257). No unexpected toxicities were observed. In the trastuzumab- containing arm, single cases of congestive heart failure, tachyarrhythmia, hypertension, and a drop to below 40% or a 10% decline from baseline in left ventricular ejection fraction were observed.

### 3.2 Other Targeted Strategies for Treatment Beyond Progression

A number of other agents targeting her2 and other members of the epidermal growth factor receptor family have been evaluated as single agents or in combination with other chemotherapy partners. These include lapatinib and pertuzumab.

The role of lapatinib, a tyrosine kinase inhibitor that inhibits both her1 and her2, was investigated in a prospective randomized phase iii clinical trial. Women with her2-positive mbc who had progressed on trastuzumab (*n* = 324) and who had received anthracyclines and taxanes, were randomized to receive capecitabine alone (2500 mg/m^2^ days 1–14, every 3 weeks) or capecitabine (2000 mg/m^2^ days 1–14, every 3 weeks) with lapatinib (1250 mg/m^2^ twice daily, days 1–14, every 3 weeks) 40,41. The primary endpoint was time to progression. Secondary endpoints included progression- free survival, overall survival, clinical benefit, partial response rate, and safety. Women assigned to the combination arm experienced a significantly longer time to progression (27 weeks vs. 19 weeks, *p* = 0.00013) than did those taking capecitabine alone. Overall response rates were higher in the combination arm (24% vs. 14%, *p* = 0.017). The hazard ratio for overall survival in the combination arm (versus capecitabine alone) was 0.78 (95% confidence interval: 0.55 to 1.12; *p* = 0.177; Table VI). Based on these data, the combination of lapatinib with capecitabine was approved by the U.S. Food and Drug Administration for patients with advanced or metastatic her2-positive breast cancer who have received prior therapy with an anthracycline, a taxane, and trastuzumab.

Lapatinib was recently approved by Health Canada (May 14, 2009) and is indicated in combination with capecitabine for the treatment of patients with advanced or metastatic breast cancer whose tumours overexpress her2. Eligible patients should have progressed on taxanes, anthracyclines, and trastuzumab before the start of the lapatinib and capecitabine combination. Health Canada approval was based on improvement in time to progression; no significant improvement in overall survival was observed.

O’Shaughnessy *et al.* examined trastuzumab and lapatinib combinations versus lapatinib alone in women with heavily pretreated her2-positive mbc (*n* = 296) progressing on trastuzumab therapy. The primary endpoint was progression-free survival; the secondary endpoints were clinical benefit rate at 24 weeks, response rate, and overall survival. Patients in the intent-to-treat population who received combination therapy experienced a significantly longer median progression-free survival (12.0 weeks vs. 8.4 weeks, *p* = 0.029) and a significantly better clinical benefit rate (25.2% vs. 13.2%, *p* = 0.02). The differences in response rate (10.3% vs. 6.9%, *p* = 0.46) and median overall survival time (51.6 weeks vs. 39.0 weeks, *p* = 0.106) were not statistically significant 42.

Pertuzumab, another humanized monoclonal antibody developed to target a different domain of the her2 receptor, has been studied in combination with trastuzumab for patients with her2-positive mbc and disease progression on, or subsequent to, trastuzumab therapy with two or more prior chemotherapy regimens 43–46. In a phase ii open-label single-arm study, a clinical benefit rate of 50.0% and an objective response rate of 24.2% (including 16.7% partial response and 7.6% complete response) were observed in 66 evaluable patients 46. No clinically significant cardiac events were observed.

### 3.3 MBC After Adjuvant Trastuzumab (Re-treatment)

There are currently no published clinical trials to guide clinicians in the management of women with her2-positive mbc who relapse after receiving adjuvant trastuzumab. A number of actively recruiting phase ii and iii studies are addressing this important clinical issue.

The rhea (Retreatment After Herceptin Adjuvant) study is a nonrandomized phase ii trial that is examining the efficacy of trastuzumab therapy alone (cohort A) or with docetaxel or paclitaxel (cohort B) in patients who have relapsed 6 months or more after completion of at least 10 months of adjuvant trastuzumab. The choice of cohort will be made for each patient by the investigator, in accordance with the investigator’s clinical practice. The planned size for each cohort is 40 patients 47.

Three ongoing phase iii trials are evaluating first-line systemic therapy regimens for women with her2-positive mbc who may or may not have received adjuvant trastuzumab. Although these trials do not specifically address the potential benefit of the addition of trastuzumab to the chemotherapy regimen (versus the absence of trastuzumab), the results of such studies may provide some insight into the role of targeted therapy in this setting.

The cleopatra trial is examining the efficacy and safety of trastuzumab and docetaxel with or without pertuzumab as first-line chemo-biologic therapy in patients with her2-positive mbc, with a disease-free interval of 12 months or more after adjuvant trastuzumab. The primary endpoint is progression-free survival; secondary endpoints are overall survival, response rate, and safety. The targeted sample size is 800 48.

The averel trial is designed to assess the efficacy and safety of first-line trastuzumab and docetaxel with or without bevacizumab in patients with her2-positive locally recurrent or metastatic breast cancer who have not received prior chemotherapy or radiotherapy for their metastatic disease. Patients will be stratified by prior adjuvant and neoadjuvant taxane chemotherapy and prior adjuvant trastuzumab therapy. Patients will be stratified by prior adjuvant or neoadjuvant taxane chemotherapy and prior adjuvant trastuzumab therapy. Patients must have completed adjuvant trastuzumab therapy 6 months or more before enrolment. The primary endpoint is progression-free survival; secondary endpoints are overall survival, overall response, duration of response, time to treatment failure, quality of life, and safety. The target sample size is 410 49.

The ma.31 (complete) trial being undertaken by the National Cancer Institute of Canada Clinical Trials Group is an international phase iii trial examining the efficacy and safety of first-line taxane-based chemotherapy combined with lapatinib (or trastuzumab) in patients with her2-positive metastatic disease. Patients are required to have had at least a 12-month interval from prior chemotherapy or her2-targeted therapy in the adjuvant or neoadjuvant setting. The primary endpoint is progression-free survival; secondary endpoints include overall survival, incidence rates of and time to central nervous system metastases at the time of progression, overall response rate, clinical benefit response rate, adverse event profile, quality of life, clinical outcomes using biomarkers, and health economics (including healthcare utilization and health utilities). The target sample size is 600 50.

### 3.4 Other Targeted Therapies

A variety of other novel targeted therapies are currently under investigation in women with her2-overexpressing mbc who may have received adjuvant trastuzumab-based therapy (that is, those who present with *de novo* metastatic disease) or for women who have progressed while receiving trastuzumab-based therapy for metastatic disease (second- or subsequent-line setting). In a phase ii trial, trastuzumab-mcc-dm1, a toxin-conjugated version of trastuzumab 51, appears to offer improved efficacy and reduced toxicity over unconjugated trastuzumab in heavily pre-treated patients 52. An ongoing phase iii trial is currently evaluating trastuzumab- mcc-dm1 in comparison with the combination of capecitabine and lapatinib in patients with her2-positive locally advanced or metastatic breast cancer who have received prior trastuzumab-based therapy 53.

The mammalian target of rapamycin, mtor, is a central regulator of G1 cell-cycle protein synthesis that precedes commitment to normal cellular replication. Inhibition of mtor has been shown to have antiproliferative activity in breast cancer by deregulation of the pi3k/Akt pathway 54–56. One mtor inhibitor, deforolimus, is now being tested in combination with trastuzumab in a single-arm phase ii trial involving her2-positive trastuzumab-refractory mbc patients. For eligible patients, at least 4 weeks must have elapsed since earlier investigational therapy, chemotherapy, or radiotherapy 57. The trio-018 international phase iii study is evaluating the addition of everolimus (or placebo) to standard trastuzumab and weekly paclitaxel in the treatment of her2-positive advanced breast cancer 58.

Histone deacetylase is another anticancer target that controls gene expression through transcription regulation 59,60. Panobinostat, an inhibitor of histone deacetylase 61, is being studied in a single-arm phase iib/iia trial in combination with trastuzumab for her2-positive mbc patients who have progressed during or after trastuzumab treatment 62. Panobinostat is also being studied as monotherapy in the trio-016 study 63.

Heat shock protein 90 (Hsp90) is a chaperone protein that enables cancer cell survival 64. The Hsp90 inhibitor IPI-504 is being investigated in a single-arm phase ii trial in combination with trastuzumab for patients with pretreated, locally advanced, or metastatic her2-positive breast cancer. Patients must have received at least two earlier regimens, with trastuzumab being a component in at least one (not including adjuvant regimens, unless progression on adjuvant treatment occurred) 65.

The selective angiopoietin 1 and 2–neutralizing “peptibody” AMG 386 inhibits angiogenesis by preventing interaction of angiopoietins with Tie2 receptors 66. This agent is currently being studied in a phase i setting combining escalating doses of AMG 386 with paclitaxel and trastuzumab, and escalating doses of AMG 386 with capecitabine and lapatinib 67.

## 4. DISCUSSION

### 4.1 Treatment Beyond Progression

Although retrospective case–cohort and prospective phase ii clinical trials (Level 3 evidence) have demonstrated safety and suggested possible clinical benefit from the continuation of trastuzumab beyond progression after first-line therapy in women with her2-positive mbc, it has been impossible to support the use of this strategy without higher quality (Level 1) evidence. The uncertainty of this therapeutic approach has been compounded by a lack of understanding of the mechanism of resistance to targeted therapies in her2-positive mbc. At the present time, a good definition of resistance from a mechanistic point of view is not available, leaving practitioners to rely on a clinical definition based on disease progression in relation to the targeted therapy. This area remains one of active, ongoing research.

The prospective randomized controlled trial conducted by the German Breast Group (gbg 26) demonstrated the clinical benefit of continuing trastuzumab with another chemotherapy partner (time to progression: 8.2 months vs. 5.6 months) 39. The absence of an overall survival advantage should not be a deterrent to the use of trastuzumab beyond first-line progression, because time to progression is an endpoint worthy of consideration. In addition, given that gbg 26 was prematurely stopped and that the targeted sample size was not reached, it is possible that the study was underpowered to detect a survival difference.

Two randomized phase iii trials have examined the role of combining (lapatinib–trastuzumab) or switching (trastuzumab to lapatinib plus capecitabine) targeted therapies in women with her2-positive mbc. Although these two studies do not directly address the question of continuing trastuzumab beyond progression (no arm without trastuzumab), the results support the contention that, in this population, continuing an anti-her2 targeted therapy may be reasonable.

#### Panel Suggestion

It is highly unlikely that additional studies examining the specific role of trastuzumab combined with “second-line” chemotherapy will be forthcoming for women with her2-positive mbc who have progressed on trastuzumab. The available evidence, including data from prospective randomized controlled trials, seems to support the continuation of her2-targeted therapy with trastuzumab or with lapatinib in combination with capecitabine. Despite these panel suggestions, the modest clinical benefits observed with this approach should encourage further research and participation in clinical trials for this patient population.

### 4.2 Re-treatment

No randomized clinical data support the use of trastuzumab or other anti-her2 therapies in women with her2-positive mbc who have received trastuzumab in the adjuvant setting. Several clinical trials are currently addressing this important clinical issue.

#### Panel Suggestion

In the absence of data, and given the benefits seen in mbc patients who continue trastuzumab beyond progression, it would seem reasonable to consider re-treatment for patients exposed to adjuvant trastuzumab who have relapsed 6 months or more after adjuvant therapy. Participation in clinical trials for this patient population is encouraged.

## 5. CONCLUSIONS

The authors of this manuscript developed treatment suggestions based on a review of the literature regarding treatment options for women with her2-positive mbc. The role of targeted therapies in this patient population will continue to evolve with the completion and publication of ongoing clinical trials.

### 5.1 Date of Author Suggestions

The panel suggestions were completed in March 2009.

## Figures and Tables

**TABLE I:** Efficacy results from pivotal first-line metastatic breast cancer

Clinical endpoint	Trial arms Slamon *et al*., 2001^18^ (*n*=469)		Trial arms Marty et al., 2005 and 2006^19^,^20^ (*n*=186)	
	Trastuzumab AND [paclitaxel OR (anthracycline AND cyclophosphamide)]	Paclitaxel OR (anthracycline AND cyclophosphamide)	Trastuzumab AND docetaxel	Docetaxel alone
Median orr (%)	50	32	61	34
	*p<*0.001		*p=*0.0002	
Median dr (months)	9.1	6.1	11.7	5.7
	*p<*0.001		*p=*0.009	
Median ttp (months)	4.7	4.6	11.7	6.1
	*p<*0.001		*p=*0.0001	
Median os (months)	25.1	20.3	31.2	22.7
	*p=*0.046		*p=*0.0325^a^	

a Trial survival results were updated at the 2006 San Antonio Breast Cancer Symposium. The superior survival benefit for trastuzumab docetaxel over docetaxel alone was maintained (31.2 months vs. 22.7 months); however, the survival difference was no longer significant (*p* = 0.0876), perhaps because most of the patients randomized to docetaxel alone (57%) subsequently crossed over to receive trastuzumab.

orr = objective response rate; dr = duration of response; ttp = time to progression; os = overall survival.

**TABLE II:** Response rates of trastuzumab in combination with chemotherapy in women with metastatic breast cancer progressing on trastuzumab: retrospective and case–cohort studies

Reference	Patients^a^ (*n*)	Therapy line							
		Second		Third		Fourth		Fifth	
		orr (%)	ttp (months)	orr (%)	ttp (months)	orr (%)	ttp (months)	orr (%)	ttp (months)
Tokajuk *et al.,* 2006^21^	14	50	5.1	—	—	—	—	—	—
		(docetaxel, vinorelbine, cisplatin, capecitabine, etoposide, gemcitabine, and trastuzumab)							
Stemmler *et al.,* 2005^22^	23	39.1	—	—	—	—	—	—	—
		(taxane, vinorelbine, or other chemotherapy, and trastuzumab)							
Gelmon *et al.,* 2004^23^	65	32	—	—	—	—	—	—	—
		(taxane, or vinorelbine and trastuzumab, or trastuzumab alone)							
García-Saénz *et al.,* 2006^24^	47 (2nd line)	29.8	4	38.1	4	20	4	0^b^	nr
	21 (3rd line)								
	10 (4th line)								
		(chemotherapy and trastuzumab)							
Metro *et al.,* 2007^25^	37 (2nd line)	29	6.7	0	4	0	4.5	—	—
	16 (3rd line)								
	9 (4th line)								
		(chemotherapy and trastuzumab)							
Fountzilas *et al.,* 2003^26^	80 (2nd line)	23.8	5.2	14.3	3.5	19.2	4.9	8.3	3.9
	49 (3rd line)								
	26 (4th line)								
	12 (5th line)								
		(vinorelbine or gemcitabine and trastuzumab)							
Adamo *et al.,* 2007^27^	26 (2nd line)	23	9	22	9^c^	—	—	—	—
	9 (3rd line)								
		(vinorelbine, taxane, pegylated liposomal doxorubicin, or gemcitabine and trastuzumab)							
Montemurro *et al.,* 2006^28^	40	18	—	—	—	—	—	—	—
		(vinorelbine, taxanes, liposomal doxorubicin, capecitabine, letrozole, or tamoxifen and trastuzumab)							
Fabi *et al.,* 2008^29^	59	27	6.7	—	—	—	—	—	—
		(chemotherapy and trastuzumab)							

a Those who received trastuzumab treatment beyond progression (in the second line, unless otherwise stated).

b Stable disease 60%.

c Reported for third-line treatment and beyond.

orr = overall response rate; ttp = time to progression; nr = not reported.

**TABLE III:** Response rates for trastuzumab in combination with chemotherapy in women with metastatic breast cancer progressing on trastuzumab, prospective phase ii trials

Reference	Patients^a^ (*n*)	Therapy line			
		Second		Third	
		orr (%)	ttp (months)	orr (%)	ttp (months)
Tripathy *et al.,* 2004^30^	93	11	—	—	—
		(chemotherapy and trastuzumab)			
Morabito *et al.,* 2006^31^	7	29	—	—	—
		(vinorelbine, gemcitabine, and trastuzumab)			
Orlando *et al.,* 2006^32^	11	18	6	—	—
		(cyclophosphamide, methotrexate, and trastuzumab)			
Del Bianco *et al.,* 2006^33^	8 (2nd line)	50	8	62.5	9
	8 (3rd line)				
		(chemotherapy and trastuzumab)			
Bartsch *et al.,* 2007^34^	40	20^b^	8^b^	—	—
		(capecitabine and trastuzumab)			
Bachelot *et al.,* 2007^35^	17	29	—	—	—
		(vinorelbine and trastuzumab)			
Modi *et al.,* 2007^36^	20	25	—	—	—
		(tanespimycin and trastuzumab)			

a Those who received trastuzumab treatment beyond progression (in the second line, unless otherwise stated).

b Reported for second-line treatment and beyond.

orr = overall response rate; ttp = time to progression.

**TABLE IV:** Summary of efficacy data from French Hermine cohort study^38^

	Efficacy endpoint	Therapy line			
		First (*n*= 177^a^)		Second (*n*=117)	
		Continued trastuzumab (*n*=107)	Discontinued trastuzumab (*n*=70)	Continued trastuzumab (*n*=87)	Discontinued trastuzumab (*n*=30)
Median os (months)	From initiation of trastuzumab	Not reached^b^	16.8	27.2	15.6
	From date of progression	21.3	4.6	15.5	11
os at 2 years (%)	From initiation of trastuzumab	73.7	24.7	55.7	41.8

a Follow-up data are available for only 177 of the 221 patients who received first-line trastuzumab-based

b After a median follow-up of 27.8 months.

os = overall survival.

**Table V:** Summary of efficacy data from the German Breast Group 26 trial

Clinical endpoint	Trial arms von Minckwitz *et al*., 2009^39^ (*n*=152)	
	Trastuzumab AND capecitabine	Capecitabine alone
ttp (months)	8.2	5.6
	*p=*0.0338	
orr (%)	48.1	27.0
	*p=*0.0115	
cbr (%)	75.3	54.0
	*p=*0.0068	
os (months)	25.5	20.4
	*p=*0.257	

ttp = time to progression; orr = overall response rate; cbr = clinical benefit rate; os = overall survival.

**TABLE VI:** Summary of efficacy data from trials by Geyer *et al.*

Clinical endpoint	Trial arms Geyer *et al*., 2006 and 2007^40^,^41^ (*n*=324)	
	Lapatinib AND capecitabine	Capecitabine alone
ttp (weeks)	27^a^	19^a^
	*p=*0.00013^a^	
pfs (%)	8.4	4.1
	*p<*0.001	
orr (%)	24^a^	14^a^
	*p=*0.017^a^	
cbr (%)	44	29
	nr	
os (months)	nr	nr
	*p=*0.177^a^	

a Based on updated data presented in 2007.

ttp = time to progression; pfs = progression-free survival; orr = objective response rate; cbr = clinical benefit rate; nr = not reported; os = overall survival.

## Footnotes

a Migliaccio I, Gutierrez MC, Wu MF, *et al.* PI3 kinase activation and response to trastuzumab or lapatinib in her-2 overexpressing locally advanced breast cancer (labc). Presented at the 31st Annual San Antonio Breast Cancer Symposium; San Antonio, TX; December 10–14, 2008.
