# Erratum

**Published:** 2009-09

Please be advised that there is an error in Table I of “The Role of HER2-targeted Therapies in Women with HER2-Overexpressing Metastatic Breast Cancer” by Dent *et al.* which appeared in *Current Oncology* Volume 16, Number 4 on page 236 in print, and page 26 online. The median ttp in the Slamon *et al*. trial in the column labeled “*Trastuzumab AND [paclitaxel OR (anthracycline AND cyclophosphamide)]”* should read 7.4 not 4.7 as originally printed. The corrected version of the table can be found below.

## Figures and Tables

**TABLE I:** Efficacy results from pivotal first-line metastatic breast cancer trials

Clinical endpoint	Trial arms Slamon et al., 2001^18^(*n*=469)		Trial arms Marty et al., 2005 and 2006^19,20^ (*n*=186)	
	Trastuzumab AND [paclitaxel OR (anthracycline AND cyclophosphamide)]	Paclitaxel OR (anthracycline AND cyclophosphamide)	Trastuzumab AND docetaxel	Docetaxel alone
Median orr (%)	50	32	61	34
	*p*<0.001		*p*=0.0002	
Median dr (months)	9.1	6.1	11.7	5.7
	*p*<0.001		*p*=0.009	
Median ttp (months)	7.4	4.6	11.7	6.1
	*p*<0.001		*p*=0.0001	
Median os (months)	25.1	20.3	31.2	22.7
	*p*=0.046		*p*=0.0325^a^	

a Trial survival results were updated at the 2006 San Antonio Breast Cancer Symposium. The superior survival benefit for trastuzumab plus docetaxel over docetaxel alone was maintained (31.2 months vs. 22.7 months); however, the survival difference was no longer significant (*p* = 0.0876), perhaps because most of the patients randomized to docetaxel alone (57%) subsequently crossed over to receive trastuzumab.

orr = objective response rate; dr = duration of response; ttp = time to progression; os = overall survival.

